# Value of Rest

**Published:** 1889-01

**Authors:** 


### VALUE OF REST.

There is no better preventive of nervous exhaustion than regular, unhurried muscular exercise (says an exchange). If we could moderate our hurry, lessen our worry and increase our open air exercise, a large proportion of nervous diseases would be abolished. For those who cannot get a sufficient holiday the best substitute is an occasional day in bed. Many whose nerves are constantly strained in their daily vocation have discovered this for themselves. A Spanish merchant in Barcelona told his medical man that he always went to bed for two or three days, whenever he could be spared from his business, and he laughed at those who spent their holidays on toilsome mountains.

One of the hardest working women in England, who has for many years conducted a large wholesale business, retains excellent nerves at an advanced age, owing, it is believed, to the habit of taking one day a week in bed. If we cannot avoid frequent agitation, we ought, if possible, to give the nervous system time to recover itself between the shocks. Even an hour's seclusion after lunch will deprive a hurried, anxious day of much of its injury. The nerves can often be overcome by stratagem when they refuse to be controlled by strength of will.—*Boston Journal of Health.*

